# Anatomy of a demand shock: Quantitative analysis of crowding in hospital emergency departments in Victoria, Australia during the 2009 influenza pandemic

**DOI:** 10.1371/journal.pone.0222851

**Published:** 2019-09-24

**Authors:** Peter Sivey, Richard McAllister, Hassan Vally, Anna Burgess, Anne-Maree Kelly

**Affiliations:** 1 School of Economics, Finance and Marketing, RMIT University, Melbourne, Victoria, Australia; 2 Department of Education and Training, Australian Government, Canberra, ACT, Australia; 3 Department of Public Health, La Trobe University, Melbourne, Victoria, Australia; 4 Department of Health and Human Services (Victoria), Melbourne, Victoria, Australia; 5 Joseph Epstein Centre for Emergency Medicine Research at Western Health and School of Medicine-Western Clinical School, The University of Melbourne, Parkville, Victoria, Australia; Azienda Ospedaliero Universitaria Careggi, ITALY

## Abstract

**Objective:**

An infectious disease outbreak such as the 2009 influenza pandemic is an unexpected demand shock to hospital emergency departments (EDs). We analysed changes in key performance metrics in (EDs) in Victoria during this pandemic to assess the impact of this demand shock.

**Design and setting:**

Descriptive time-series analysis and longitudinal regression analysis of data from the Victorian Emergency Minimum Dataset (VEMD) using data from the 38 EDs that submit data to the state’s Department of Health and Human Services.

**Main outcome measures:**

Daily number of presentations, influenza-like-illness (ILI) presentations, daily mean waiting time (time to first being seen by a doctor), daily number of patients who did-not-wait and daily number of access-blocked patients (admitted patients with length of stay >8 hours) at a system and hospital-level.

**Results:**

During the influenza pandemic, mean waiting time increased by up to 25%, access block increased by 32% and did not wait presentations increased by 69% above pre-pandemic levels. The peaks of all three crowding variables corresponded approximately to the peak in admitted ILI presentations. Longitudinal fixed-effects regression analysis estimated positive and statistically significant associations between mean waiting times, did not wait presentations and access block and ILI presentations.

**Conclusions:**

This pandemic event caused excess demand leading to increased waiting times, did-not-wait patients and access block. Increases in admitted patients were more strongly associated with crowding than non-admitted patients during the pandemic period, so policies to divert or mitigate low-complexity non-admitted patients are unlikely to be effective in reducing ED crowding.

## Introduction

In Victoria, emergency departments (EDs) provide care free-of-charge for patients who present with urgent health problems. Demand for emergency care varies substantially from month to month, week to week and day to day. This can leave EDs exposed to large unanticipated surges in demand, particularly those caused by external events in the community. Events which can cause large demand surges include outbreaks of infectious disease [1, 2] or weather events such as heatwaves [3] or thunderstorm asthma [4]. Unanticipated demand surges are significant because EDs may not be able to adjust their inputs (e.g. staffing and facilities) in an appropriate time frame so will have to cope with the extra demand within existing constraints. In this study, data which highlights the system-level constraints of hospital EDs were analysed to give insight into how hospital EDs cope with demand surges.

Studies have considered the determinants of ED waiting times [5] and ED crowding [6] including the role of access block [7, 8], and the relationship between waiting time and patients who did not wait for treatment [9]. However these relationships have usually been studied in a general context. Studies that have focused on particular demand surges such as the influenza pandemic or heat waves have focused on surveillance data [10–12], health outcomes [13, 14], costs [15] or particular groups of patients such as children [2]. There is a lack of studies looking at ED crowding outcomes in a pandemic, apart from one using survey outcome measures [16].

The aims of this project were to analyse changes in waiting times, access block and did-not-wait patients in Victorian EDs during the 2009 influenza pandemic. In this paper ‘crowding’ outcome variables are analysed over the time period of a particular demand ‘shock.’ The advantage of this approach over previous research is that the demand shock can be regarded as ‘exogenous’–unanticipated and unrelated to supply and organisation of care at hospitals. This allows our results to better represent the likely causal relationship between the extra demand that hospitals faced during this period and the crowding outcomes.

The 2009 influenza pandemic was characterised by a sharp increase in influenza like illness (ILI) presentations at hospitals in Victoria much earlier in the year than usual (from the middle of May). Studies have shown this pandemic caused much greater increase in hospital and ICU admissions [12, 17] than the average yearly influenza season.

This study builds upon a literature in health economics which has sought to explain ‘crowding’ variables such as waiting times as a function of the gap between demand and supply [18–20]. We also contribute to the understanding of whether low- or high-complexity presentations are primary responsible for crowding outcomes. Whereas the role of low-complexity patients (for example non-admitted patients) has been questioned in the past [21, 22], we are the first to analyse this issue in the context of a demand shock to the ED.

## Methods

### Study design

This was a descriptive time-series analysis and longitudinal regression analysis of data from the Victorian Emergency Minimum Dataset (VEMD). The VEMD is a de-identified set of patient-level data reported by hospitals to the Victorian Department of Health and Human Services. It covers all presentations at the 38 public EDs which are required to submit data to the department. We used the reference period 16 April to 29 August 2009 including 521,988 presentations. This reference period covers the winter of 2009 including the usual time period for the influenza season. The outcomes of interest were influenza-like-illness (ILI) presentations and ED metrics including waiting times, access block and did-not-wait patients.

For this study, ILI was defined broadly in order to maximise sensitivity [23, 24]. Our rationale is that previous studies have found that ILI defined with International Classification of Disease [ICD] 10 codes imperfectly correlates with other measures of influenza such as notified cases [24]. In the context of this study the choice was made to maximise sensitivity to make sure any trends in influenza cases were fully captured in the definition used. ILI presentations are therefore defined to include patients whose primary diagnosis is in the ICD10-AM codes for “fever” (R50), “headache” (R51), “virus” (B34) and all primary diagnoses in the J chapter of ICD10-AM including all respiratory diseases (including the codes for influenza J09, J10 and J11).

### Ethics

The research project was assessed as ‘negligible risk’ by the College of Arts, Social Sciences and Commerce Human Ethics Sub-Committee at La Trobe University (reference E16/3).

### Data analyses

Daily time series charts on aggregate variables over the reference period are presented. Four variables were analysed: (1a) the total number of presentations per day, (1b) total number of influenza-like-illness (ILI) presentations, and (1c) total number of admitted and (1d) non-admitted ILI presentations; (2) the waiting time for each patient (difference between the time the patient first presented at the ED and the time when they were seen by a doctor); (3) the total number of patients who ‘did not wait’; (4) the total number of patients who experienced access block (spent more than 8 hours total length of stay in the ED awaiting ward admission). Charts are presented with two series shown together on different vertical axes (1a and 1b, 1c and 1d, 2 and 3, 4) to visualise the degree of correlation between these variables over time.

For all four variables there are significant day of the week effects which lead the raw time series to show high volatility. For this reason, data on all variables is presented in time series charts as a seven-day moving average.

Additionally, we used data at the hospital/day level to conduct simple regression analysis and longitudinal fixed-effects regression analysis [25] (within-hospital estimation) of the relationship between three alternative dependent variables—mean waiting time, total did-not-wait patients, total access-blocked patients—with the single explanatory variable measuring the total daily admitted or non-admitted ILI presentations respectively.

Simple regression models use variation across hospitals and over time to identify coefficients. The longitudinal fixed-effects models control for all time-invariant hospital-level factors so only changes over time in the number of ILI presentations and the dependent variables within hospitals identify coefficients. Hausman tests were conducted comparing the fixed-effects models against their random-effects counterparts and the null hypothesis was rejected at the 5% level for four out of the six models, favouring the fixed effects approach.

Data analyses were conducted using STATA 15 [26].

## Results

**Fig 1:**
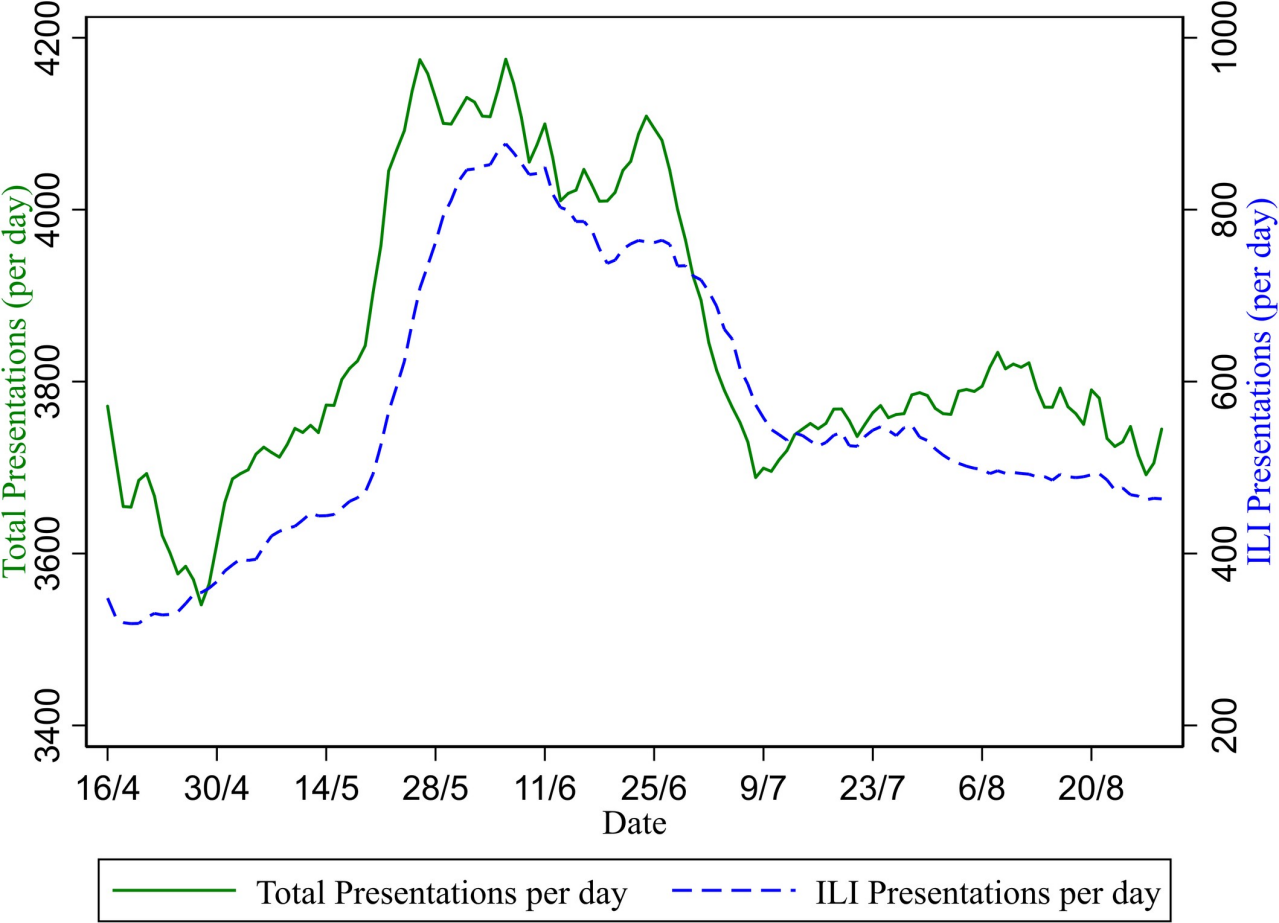
Total presentations per day and total ILI presentations per day (seven-day moving average). The increase in ILI presentations observed in May and June 2009 can be divided into patients who were admitted into the hospital (including short stay wards and ICU) and those not admitted (Fig 2). This division shows two distinct patterns: the non-admitted patients peak on 6 June at 722 per day whereas the admitted presentations peak on 24 June at 180 per day.

The seven-day moving average of total presentations per day and ILI presentations per day in late May and early June 2009 show an increase in total presentations from 3772 on 15 May 2009 to 4175 on 6 June (10.7%) and an increase in ILI presentations from 445 to 876 over the same period (Fig 1). The increase in total presentations in this time period (403) approximately corresponds to the increase in ILI presentations (431), suggesting the increase in total presentations is driven by ILI patients.

The seven-day average of did-not-wait presentations increased from 237 per day on 15 May to 317 per day on 6 June then further to 400 per day on 24 June (total increase of 69%). This increase was substantial in proportional terms as total presentations only increased approximately 10% over this period (Fig 1). Did-not-wait numbers appear to drop back to near-normal levels soon after the end of the pandemic, with the seven-day average of 250 per day on the 9 July.

### Regression analysis

**Fig 2:**
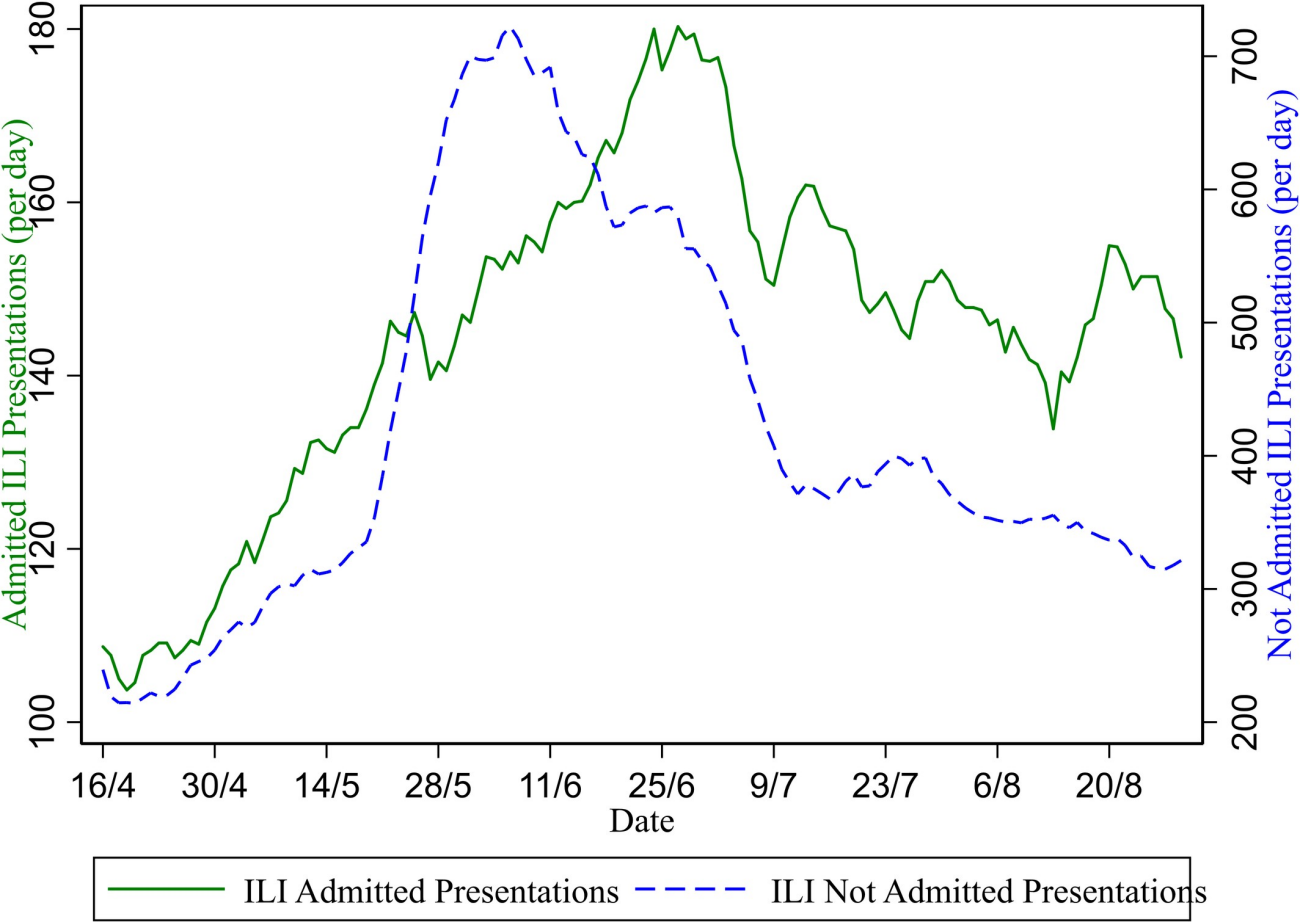
Admitted and non-admitted ILI presentations per day (seven-day moving average). Both average waiting times and the number of patients who did not wait increased markedly during the pandemic period (Fig 3). From 41.1 minutes on 15 May (before the pandemic), the seven day average waiting time rose to 43.7 minutes on 6 June (the non-admitted presentations peak) then to 52 minutes on 24 June (the admitted patients peak). Average waiting times remained elevated after the end of the pandemic, only declining gradually through the rest of the winter of 2009.

**Fig 3:**
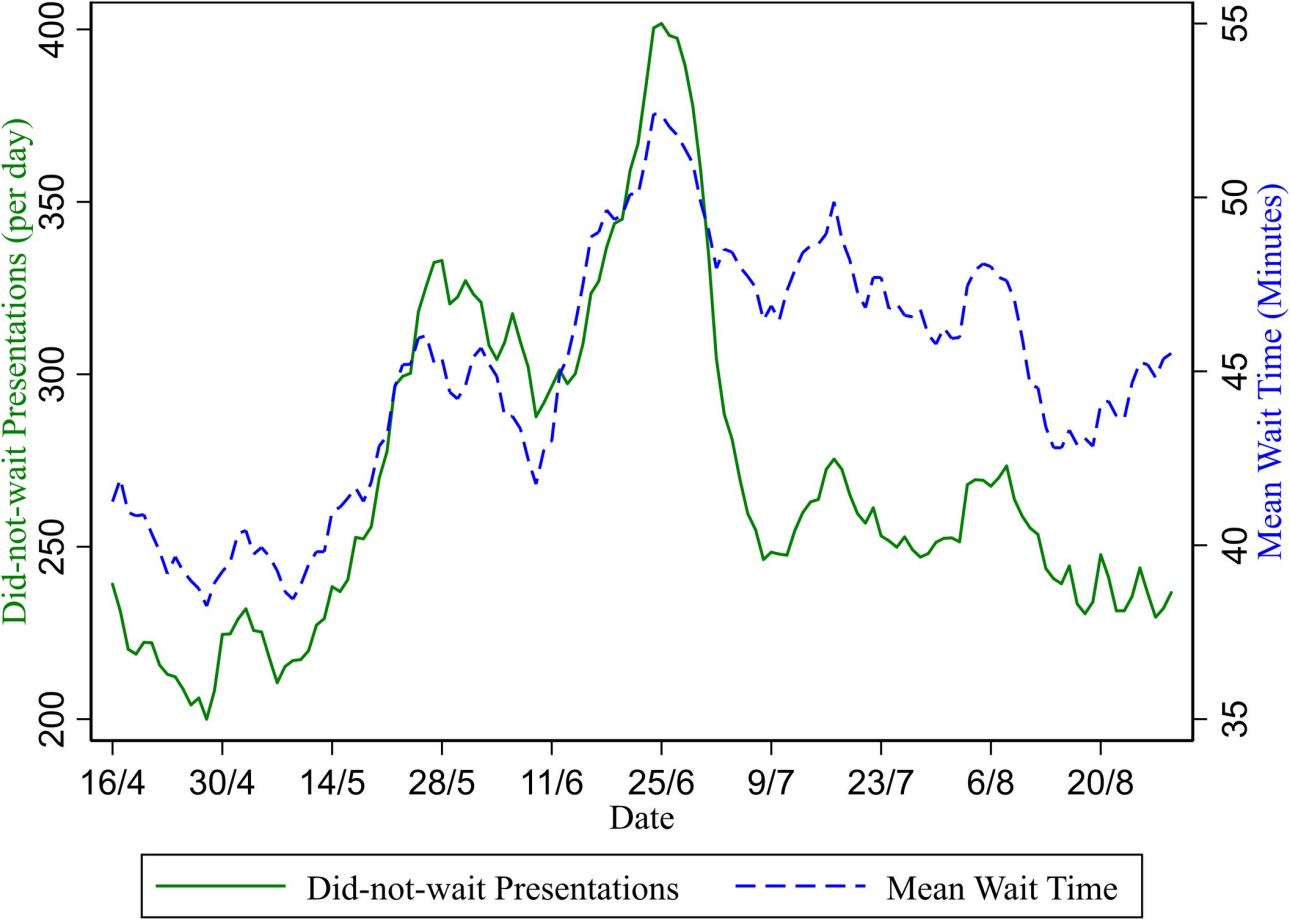
Average waiting time and number of did-not-wait presentations (seven-day moving average). The seven-day average number of patients experiencing access block (Fig 4) increases from 285 per day on 15 May up to 296 per day on 6 June and then further to 377 per day (total increase of 32%) on 24 June at the peak of admitted presentations period.

**Fig 4:**
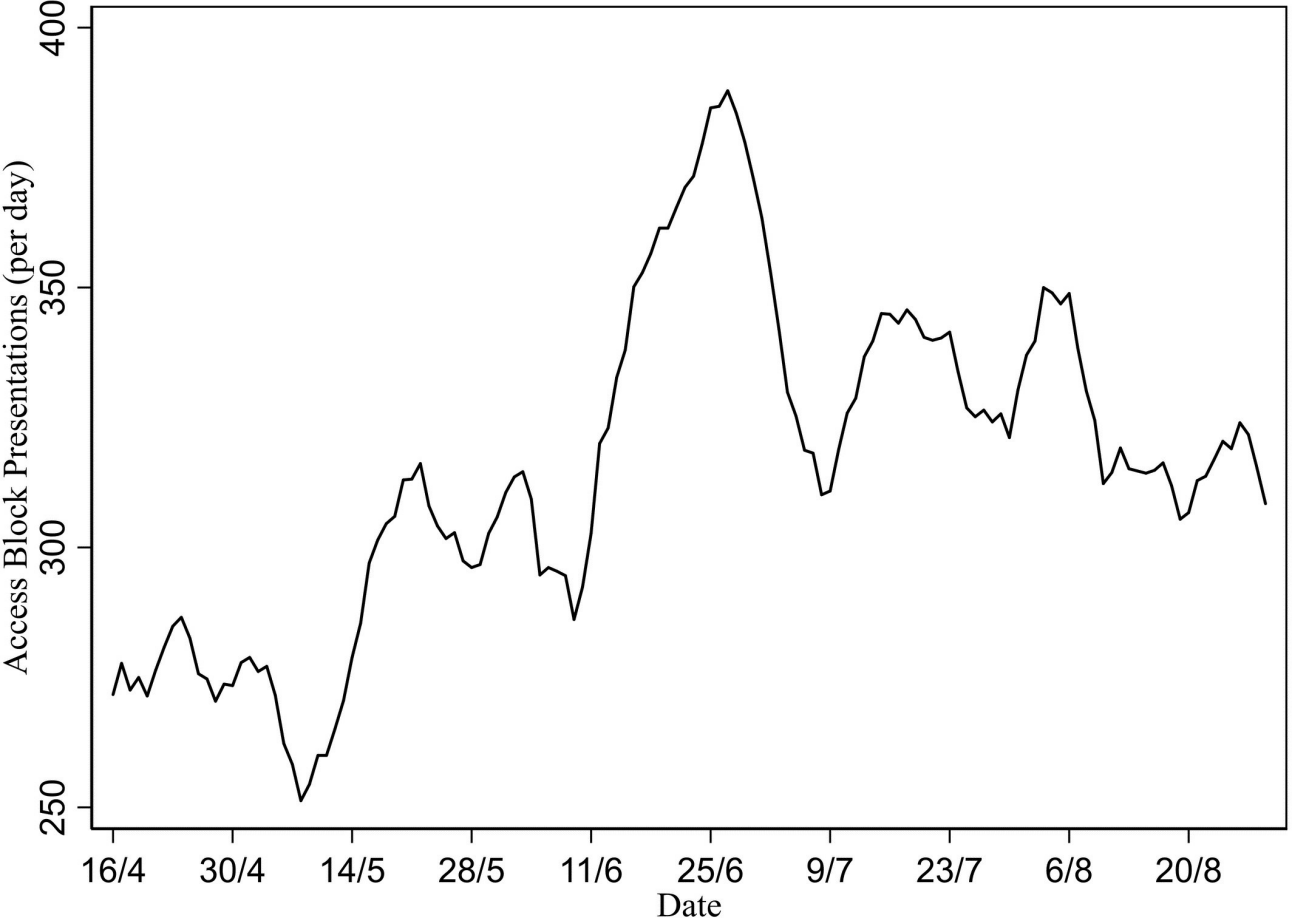
Number of access-block presentations (seven-day moving average).

The regression analysis uses data from all 38 hospitals for the reference period (136 days) giving 5,168 observations. Four different regression analyses, simple regression and longitudinal fixed effects models for both admitted and non-admitted ILI presentations, were conducted for each of three dependent variables giving 12 analyses in total (Table 1). The coefficient estimates for the simple regression models show a positive and statistically significant relationship between both the number of admitted and non-admitted ILI presentations and all three crowding variables using variation between hospitals and over time. The longitudinal fixed-effects regression models also show that within hospitals, time periods with higher admitted and non-admitted ILI presentations are statistically significantly associated with higher levels of the crowding outcomes, with the exception of the coefficient for the effect of non-admitted ILI presentations on average waiting time in the longitudinal fixed-effects model. Throughout, the crowding outcomes are more strongly associated with the admitted ILI presentations than the non-admitted ILI presentations, confirming the finding in Figs 2, 3 and 4.

**Table 1:** Regression results.

Dependent Variable ↓	Simple regression on non-admitted ILI presentations	Longitudinal fixed-effects regression (within-hospital variation) on non-admitted ILI presentations	Simple regression on admitted ILI presentations	Longitudinal fixed-effects regression (within-hospital variation) on admitted ILI presentations
Average waiting time (mins)	0.245 [0.200, 0.290]	0.166 [-0.073, 0.405]	0.884 [0.738, 1.030]	1.194 [0.893, 1.494]
Did-not-wait patients	0.467 [0.450, 0.483]	0.154 [0.063,0.245]	1.401 [1.346, 1.457]	0.429 [0.262, 0.595]
Number of access blocked patients	0.643 [0.608, 0.678]	0.179 [0.072, 0.286]	3.336 [3.249, 3.423]	0.974 [0.765, 1.184]
Sample size	5,168			

Coefficient estimates [with 95% confidence intervals] from simple linear regression and longitudinal fixed-effects regression models using hospital/day observations in period 16/04/2009 to 29/08/2009 of average waiting time, number of did-not-wait patients, and number of access-blocked patients, on non-admitted and admitted ILI presentations. Standard errors are adjusted for clustering at the hospital level.

The coefficient estimates can be interpreted as the change in the dependent variable associated with one additional non-admitted or admitted ILI presentation. For example, the first coefficient estimate in the first row suggest one additional non-admitted ILI presentation is associated with a 0.245 minute increase in average waiting time in the simple regression model. The final two columns of the first row show that one additional admitted ILI presentation is associated with a 0.884 minute or 1.194 minute increase in average waiting time in the simple and longitudinal models respectively. A notable result is that one additional admitted ILI presentation is associated with 0.429 additional did-not-wait patients and 0.974 additional access blocked patients in the longitudinal models.

As the number of ILI patients more than doubled in the pandemic period, increasing from 445 per day to 876 per day these coefficient estimates predict substantial impacts on crowding at the hospital-level associated with the pandemic.

## Discussion

Outbreaks of infectious disease such as the 2009 influenza pandemic provide ‘natural experiments’ to test the impact of demand surges on EDs. The performance of hospitals in response can be observed in terms of waiting times, did-not-waits and access block. Total daily presentations increased by approximately 10% during the pandemic period with most of the increase due to ILI, defined broadly. These findings may inform planning for future disease outbreaks or other demand shocks. The results may also provide some lessons in terms of how these variables react on a day-to-day basis to smaller or more localised demand shocks.

This study found a substantial and sustained increase in waiting time (approximately 25%) over the period of the pandemic. It also found a substantial increase in the number of did-not wait patients, which may be attributable to patients reacting to higher waiting time by choosing to leave the ED without treatment and seek alternative health care. Additionally, there was a substantial increase in ‘access block’ (approximately 32%), indicating the lack of availability of ward beds for patients being admitted from the ED. This is another symptom of excess demand which may particularly impact patients with high care needs requiring hospital admission.

The type of presentation affects the impact on crowding outcomes (Fig 3). Whereas there is an early peak in the number of non-admitted ILI presentations (6 June), the number of admitted ILI presentations peaks later on 24 June (a finding also mirrored in ICU admissions [12]). It is evident from Figs 3 and 4 that this later peak in admitted patients is a more significant determinant of the deterioration in waiting time and access blocked patients. This result suggests high-complexity (admitted) patients may be more important as determinants of crowding than low-complexity (non-admitted) patients. One possible caveat is that although the peak of did-not-wait patients also matches the admitted patients peak around 24 June, the pattern of changes over time for did-not-wait patients more closely matches non-admitted patients. For example, the rapid fall in did-not-wait patients after 24 June closely resembles a similar rapid fall in non-admitted patients. It is plausible that low complexity (non-admitted) patients are more likely to be those who could choose to leave the ED without being seen when waiting times are high.

The longitudinal regression analysis provides a more robust test of the association between crowding outcomes and admitted ILI presentations. Coefficient estimates of 1.194, 0.429 and 0.974 are estimated for the association between average waiting time (in mins), access blocked patients, and did-not wait patients with admitted ILI patients at the hospital-level and are larger than the effects for the non-admitted presentations. The associations shown at the aggregate level in Figs 2 to 4 are consistent with within-hospital (fixed effects) longitudinal regression analysis. Individual hospitals who had more ILI patients requiring admission suffered more with respect to waiting time, access block and did-not-wait patients.

The findings have suggestive implications for policy to improve the management of excess demand in EDs. Firstly, all three crowding variables were more responsive to admitted rather than non-admitted presentations and admitted patients are unlikely to be suitable for primary care management. This finding therefore casts doubt on the effectiveness of policies to ‘divert’ primary care-suitable patients away from EDs as a way of avoiding crowding, especially during demand shocks. This finding reflects some of the recent literature [21, 22] but in the context of a specific demand shock, the 2009 influenza pandemic, which provides stronger evidence than associations between hospitals or over time.

Secondly, among the outcomes analysed, we found the number of did-not-wait patients had the largest proportional increase and peaked during the peak of admitted presentations. This finding suggests patients’ responded to higher waiting times during the pandemic by reducing their demand for care (leaving before treatment). Whether this response is desirable for policymakers depends on two factors. Firstly, by restraining demand waiting times may act as an effective rationing mechanism during periods of high demand, especially if the lowest-urgency patients are deterred by higher waiting times [19]. Secondly, however, the increase in did-not-wait patients associated with higher waiting times may lead to more adverse health outcomes as patients who may have benefited from hospital care delay such treatment [27, 28].

Future research could focus on evaluating health outcomes of did-not-wait patients, especially the large increases that occur during demand shocks to gauge if further policy measures to moderate these outcomes may be necessary.

## Conclusions

The 2009 influenza pandemic caused excess demand leading to increased waiting times, did-not-wait patients and access block in EDs in Victoria, Australia. Increases in admitted patients were more strongly associated with crowding than non-admitted patients during the pandemic period, so policies to divert or mitigate non-admitted patients are unlikely to be effective in reducing ED crowding.
